# Assessment of hepatitis-related knowledge, attitudes, and practices on quality of life with the moderating role of internalized stigma among hepatitis B-positive patients in Pakistan

**DOI:** 10.1080/21642850.2023.2192782

**Published:** 2023-03-30

**Authors:** Saba Ahmed, Rosario Yslado Méndez, Shaheryar Naveed, Shoaib Akhter, Iqra Mushtaque, Mareen A. Malik, Waqar Ahmad, Roger Norabuena Figueroa, Ammar Younas

**Affiliations:** a Fatima Jinnah Women University, Rawalpindi, Pakistan; b Universidad Nacional Santiago Antunez de Mayolo, Huaraz, Perú; c Department of Sociology, University of Layyah, Layyah, Pakistan; d Department of Psychology, Quaid-e- Azam University, Islmabad, Pakistan; e Universidad Privada San Juan Bautista, Lima, Perú; f Business Law Department, Tashkent State University of Law, Tashkent, Uzbekistan

**Keywords:** Hepatitis, knowledge, management, quality of life, stigmatization

## Abstract

**Aim:**

This study aimed to assess the Pakistani hepatitis B patients’ knowledge, attitudes, and practices towards hepatitis management and the impact of self-management on the quality of life of hepatitis B patients as well as the moderating role of stigmatization.

**Methods:**

A cross-sectional study design was used, and the data was collected from a total of 432 hepatitis B positive patients through a self-designed questionnaire. The studied subjects consisted of men (*n* = 205, 47%), women (*n* = 165, 38%), and transgender (*n* = 62, 14%). The obtained data were statistically analyzed using SPSS software version 26.0 for Windows.

**Results:**

The mean age of the study participants was 48. Knowledge has a significant positive relationship with hepatitis self-management and quality of life, whereas knowledge has a negative relationship with stigmatization. Furthermore, multivariate analysis revealed that men were more knowledgeable about the disease than women and transgender people (6.14 ± 2.08 vs. 3.23 ± 1.61 vs. 1.03 ± 0.73, F = 8.2**, *p* = .000). On the scale of attitude and practice, significant gender differences were found. Women had more experience with hepatitis self-management than men or transgender (4.21 ± 13.0 vs. 2.17 ± 6.02 vs. 0.37 ± 0.31, F = 6.21**, *p* = .000). The regression analysis showed that self-management has a positive association with quality of life (B = 0.36, *p* = .001). The moderation analysis revealed that stigmatization negatively moderates the relationship between self-management and quality of life (B = −0.53, *p* = .001).

**Conclusion:**

Generally, patients had good knowledge about the disease and its self-management. However, a societal and community-level awareness campaign should be organized on the quality of life and stigmatization of people with chronic illness regarding their human rights, dignity, and physical, mental, and social well-being.

## Introduction

Hepatitis is becoming a major health issue all over the globe as it remains asymptomatic for years and leads to serious morbidity (Leoni et al., 2022). Hepatitis can result in impaired liver function, severe liver damage, and acute liver failure, all of which are potentially fatal (Stravitz & Lee, 2019; Wendon et al., 2017). According to a survey, 1.34 million deaths were attributed to viral hepatitis, similarly, 340 million new cases of acute hepatitis were reported, whereas 257 million people had chronic HBV and 71 million people had chronic HCV (James et al., 2018). Only the hepatitis B, C, and D viruses cause chronic hepatitis (World Health Organization, 2022). HBV and HCV, which are transmitted through sexual contact and injections, are responsible for the majority of viral hepatitis-related deaths (World Health Organization, 2022). Many people infected with HBV have no symptoms, yet acute and chronic infections can cause anything from nonspecific symptoms to organ failure. The most common way for babies to get HBV is through mother-to-child transmission, which occurs when HBV is passed from an infected mother to her baby during prenatal transmission (while the baby is still in the womb), natal transmission (after birth), or postnatal transmission (after the baby has been born) (during caregiving or through breast milk) (Gebrecherkos et al., 2020; Lette, 2019).

According to the World Health Organization (WHO), many countries face challenges regarding hepatitis due to a lack of knowledge about the disease (Cohen et al., 2018). In developed countries like Canada, 91% of the patients reported that knowledge regarding hepatitis is imperative to remain safe from the disease. At the same time, 50% of the people reported having poor knowledge of the disease. As the recurrence of hepatitis is expanding worldwide, attitude is considered one of the most effective ways to protect the populace’s health (Ahmad et al., 2016). A positive attitude can likewise decrease the spread of the hepatitis infection. Avoidance of any infection requires adequate knowledge, a positive attitude, and good practices (KAP) among the individuals (Shrestha et al., 2020). An increase in knowledge leads to an improvement in public attitudes and practices towards hepatitis B screening and prevention, thus reducing the prevalence of this disease in the population (Dwiartama et al., 2022). As a result, knowledge, attitude, and practice (KAP) studies focus on resolving public ambiguities and are widely used in the populace (Al-Shamiri et al., 2017).

Pakistan is the second-most affected country after Egypt (Al Kanaani et al., 2018). It is a developing country and faces many economic, technological, and literacy challenges, making it more vulnerable to contagious diseases like hepatitis (Jamil et al., 2010). On the other hand, Liu et al. (2022) reported that developing countries are more susceptible to being affected by hepatitis, and there is a need to pay more attention to this issue on the Asian continent. The scarcity of information from Pakistan and KAP about hepatitis among the populace is rarely investigated, and low-income countries require extensive management strategies and preventive measures. Therefore, this study aimed to assess hepatitis-related knowledge, attitudes, and practices and the moderating role of internalized stigma on quality of life among hepatitis B-positive patients in Pakistan.

## Literature review

### B-positive patients in Pakistan

Hepatitis is more prevalent in the territories of Africa and Asia, wherein 13.9% of the cases are found in Egypt, and it is lower in industrialized countries (Stockdale et al., 2020). For instance, 2.5% of the cases are found in Western and Northern Europe, North America, and Australia (Schmelzer et al., 2020). The examination was coordinated in Manshera (city of Pakistan) to survey the data, predominance, and knowledge with respect to HCV among inhabitants. In the study area, the overall prevalence of HCV was 10.3 percent. The positive attitude among male individuals was 11.8%, while that among female individuals was 37.9% (Brener et al., 2019; Nawaz et al., 2018).

Egypt reports the most striking recurrence in the world, with a predominance pace of more than 20% (Elbahrawy et al., 2021; Gomaa et al., 2017). An investigation conducted in the Canadian state found poor knowledge about this disease among provincial and metropolitan students (Ngami et al., 2020). One more KAP study was conducted in the United States of America to examine young adults’ knowledge of hepatitis. It was found that urban people had adequate knowledge compared to their counterparts living in villages (Rathi et al., 2018). Chronic liver disease (CLD) is the 10th factor responsible for death among grown-ups in the United States and heavy expenditure on treatment (Shindano et al., 2016). People-based examinations show that 40% of chronic liver infections are HBV-related, and people have to bear 8,000–10,000 rupees in treatment costs (Nalli et al., 2017; Rostamzadeh et al., 2018; Saquib et al., 2019).

The after-effects of a review uncover that HBV patients have poor KAP towards their sickness. Only a few patients are familiar with HBV transmission; this lack of knowledge about HBV transmission can be attributed to increased HBV recurrence (Karimi-Sari et al., 2017). This finding is in accordance with a study that was conducted before, where inadequate knowledge of various populations from various areas was found. Such findings raise grave concerns about the knowledge and perception of the general public toward hepatitis. But little research has been conducted to date (Hang Pham et al., 2019). Patients in recent studies showed poor practice towards hepatitis. A few patients showed up for hepatitis screening before they were determined to have the disease. Most patients were not adopting precautionary measures, which presented them with the risk of spreading hepatitis disease within their group of friends (Rathi et al., 2018).

### Hepatitis self-management

Self-management is essential in the treatment of chronic conditions. Self-management refers to patients’ ability to control their symptoms, treatments, lifestyle changes, and physical and psychological consequences (Hughes et al., 2017). The willingness and capability of the patient to engage in daily self-management activities is the most important factor in effective self-management . Despite the necessity of self-management, Chinese hepatitis -B patients engage in self-management activities such as follow-up visits, abstinence from alcohol, and moderate exercise less frequently (Kong et al., 2015; Xu et al., 2018).

Patients with chronic diseases can improve the daily management of their conditions through self-management education (Cui et al., 2019). Patients develop motivation to make changes in their lives as well as problem-solving abilities. Self-management education improves adherence to antiviral therapy (Shah & Abu-Amara, 2013). According to a study of HBV and/or HCV patient education studies (Shah & Abu-Amara, 2013), nurse-led sessions enhanced patients’ adherence to medication. A year after the intervention finished, self-management training improved depression and quality of life in another trial (Moriyama et al., 2013). In China, self-management education enhanced self-efficacy and quality of life (Chin et al., 2015). These studies just provided data. In China, non-adherence to HBV or HCV antiviral treatment is prevalent. In China, the lack of education regarding self-management during hospitalization for HBV or HCV and after discharge did not address patients’ concerns about adherence. We focused more on mental health issues in our program, began implementing the program while the patient was still in the hospital, and continued our educational efforts after the patient was discharged, which increased patient engagement, decreased anxiety, and prevented dropouts (Xu et al., 2018). Chronic hepatitis B affects many aspects of a patient’s life, making treatment essential. The self-management behaviors of this cohort are rarely studied (Kong et al., 2019). The association between self-management activities and illness awareness in chronic hepatitis B patients remains unknown. The development of drugs to promote self-management in chronic hepatitis B patients focuses on identifying the relationships between these characteristics and self-management techniques.

### Hepatitis effect on quality of life

Clinical researchers have recently addressed quality of life. The concept of quality of life is comprehensive, encompassing psychological, physical, and social functioning, as well as other elements that are affected by a health condition (Freeland et al., 2021). It is a crucial factor in determining health. Prior study has indicated that as the disease develops, the quality of life (QoL) of individuals with HBV declines (Lam et al., 2009). Chronic hepatitis also leads to psychological issues along with a deteriorated impact on physical health and poor quality of life (Khanam et al., 2021; Mushtaque et al., 2022). People who have hepatitis remain uncertain about the development of the disease, which causes death anxiety in them (Maqsood et al., 2021). These people experience anxiety, depression, and lack of emotional control more often than healthier people, which leads to a fear of being discriminated against and stigmatized (Nawaz et al., 2021). It aids patient management and gives healthcare decision-makers current clinical and financial information (Abdo, 2012). Patients with chronic hepatitis C may experience numerous extrahepatic symptoms, including anorexia, lethargy, myalgia, arthralgia, irritability, and headaches (Suzuki et al., 2016). Patients with Hepatitis B have a lower quality of life in southeast China. Income and disease stage have become important factors in determining the quality of life scores. Improving social and medical support may enhance the quality of life for people with hepatitis B, especially those with a low socioeconomic position and an advanced stage of the disease (Chen et al., 2021). Few studies indicate that the quality of life of patients with hepatitis B is normal or almost normal (HBV); Cardoso & Silva, 2017; Miyasaka et al., 2021).

### Discrimination and stigma faced by hepatitis patients

In various countries, patients with hepatitis B, hepatitis C, and HIV face stigma and prejudice. HIV stigma is a major obstacle to testing and treatment. The stigma associated with HBV is limited to Southeast Asia, the Western Pacific, and communities of Asian immigrants; it does not extend to non-Asian groups (Smith-Palmer et al., 2020). Numerous factors contribute to the stigma surrounding HBV. Generally, a lack of knowledge of HBV transmission channels is the cause of preconceived notions that a person utilizes drugs or is sexually promiscuous, as well as an unfounded fear of transferring the disease. The stigma associated with HIV is intersectional. Intersectional or ‘layered’ stigma results from the ‘synergistic, mutually constitutive interaction between social identities and structural injustices,’ which might be personal, societal, or structural. Increased HIV and HBV prevalence rates contribute to the stigmatization of drug users and sex workers (Rice et al., 2018).

**Figure 1.**
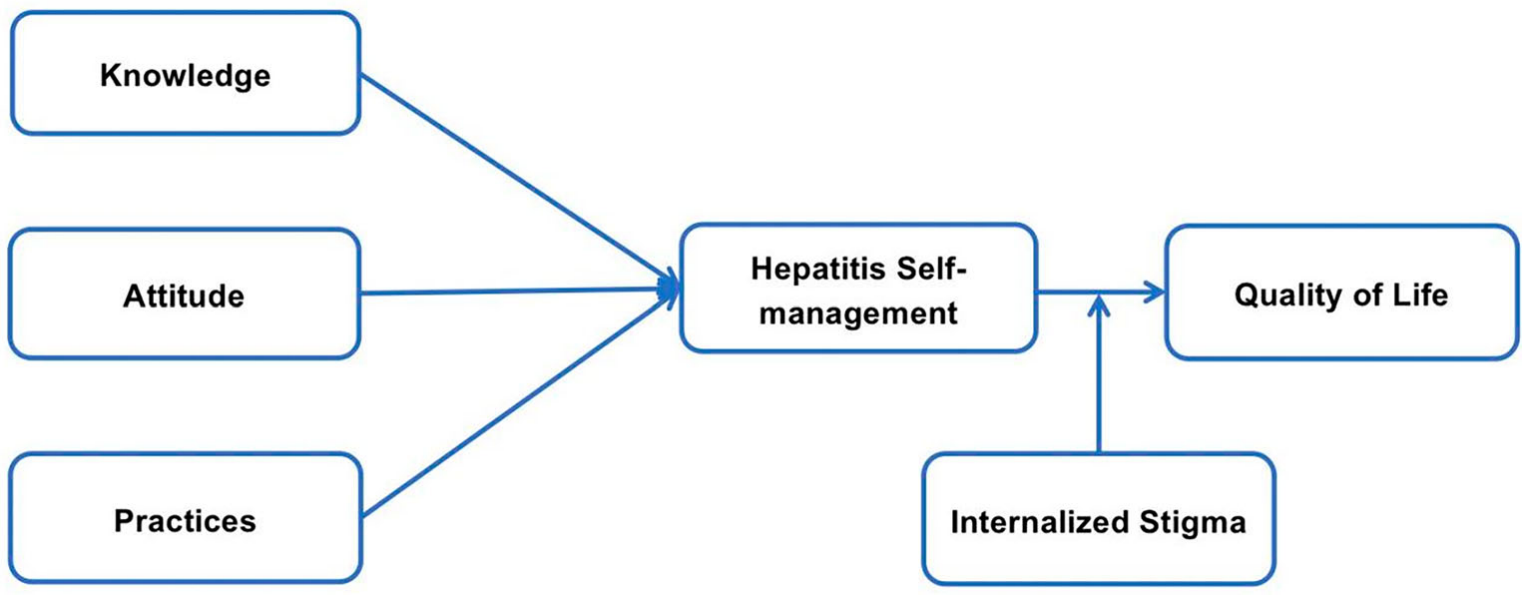
Conceptual framework.

According to van der Scheun et al. (2019), patients with hepatitis internalized stigma (self-stigma) due to feelings of shame and guilt. As hepatitis reduces the opportunities for economic benefits, education, and employment (Wallace & Milev, 2017), it worsens the social and self-stigma. People adopt various ways to cope with the disease, including denial of the disease’s existence and a healthier lifestyle (Matreja et al., 2015). These choices for a healthier lifestyle include adopting practices to reduce the conception of hepatitis. The conceptual framework is presented in Figure 1.

## Hypothesis of the research

H1: Knowledge, Attitudes and Practices towards hepatitis B significantly affect hepatitis B self-management.

H2: Hepatitis self-management predicts quality of life for hepatitis B patients.

H3: Internalizing stigma would negatively moderate hepatitis management and quality of life among hepatitis B patients.

## Methods

The study employs a quantitative method and cross-sectional research design was used. The sample size was calculated by using G power software. Convenience sampling technique was used to collect data from outpatient departments and inpatient medical wards of four main hospitals in Rawalpindi and Islamabad, including Holy Family Hospital, Benazir Bhutto Hospital, Pakistan Institute of Medical Sciences (PIMS), and Polyclinic. Patients from these facilities who volunteered to participate in the study and were undergoing hepatitis treatment were included. The patient’s data was obtained from the hospital labs and the patients’ registration desk. Afterward, with the patients’ consent, data was collected by communicating the study’s objective through a pre-designed survey. The data was inquired in two sections. The first section included information regarding demographics such as education, age, and social class; the second section inquired about the patients’ knowledge, attitudes, practices, and internalized stigma about hepatitis. The data collector offered help to those who were unable to read and write to fill out the survey.

## Ethical approval

An Ethical Approval was obtained from the research committee of Fatima Jinnah Women University and the research reference number is HEP-22-106. Informed consent was also obtained from the participants and also ensures the Helsinki Declaration and any subsequent amendments.

### Instrument of the study

1. **Participants’ information sheet:** On the participant information sheet, we obtained the participant’s general information about gender, age, level of education, duration of illness, marital status, and family history.
2. **The KAP scale:** In this study, data were gathered using the Shrestha et al. (2020) scale. It is a five-point Likert scale. The scale includes five items that assess knowledge, five items that assess attitude, and five items that assess practice toward hepatitis. The scale’s reliability is 0.784, which shows that the scale is reliable for the data collection.
3. **Hepatitis self-management scale:** In the current study, a 25 items scale was used to measure the patient’s self-management. The scale was developed by Kong et al. (2018). The scale has four dimensions (1) management of symptoms, (2) lifestyle management, (3) psycho-social coping, and (4) disease-related information management. The reliability of the scale is 0.887.
4. **WHO-Quality of life scale:** it is a 26-item scale. The scale’s reliability is 0.921 (World Health Organization, 2012). The scale has 5 dimensions (physical health, general health, environment, psychological health, and social relationship). In the current study, we examined the patients’ psychological health, social relationship, and environment to measure their quality of life.
5. **Hepatitis-related self-stigma scale:** Earnshaw and Quinn (2011) developed the self-stigma scale. It is an 11-item scale. The reliability of the scale is 0.89. It is a five-point likert scale.

### Statistical analysis

The current study used Pearson moment correlation, multivariate and regression, and moderation analysis to examine the relationship. The collected data were analyzed through SPSS software version 26.0 for Windows. *P*-values less than 0.05 were recorded as statistically significant.

## Results

A total of 432 participants voluntarily participated in the current study. Most participants were male (47%), female (38%), and transgender male (8.1%) and transgender female (6.25%) with 54% living in rural areas and 45% in urban areas. The mean age of the participants was 48 years. Most of the participants received only secondary level education (52%), some of them graduated (36%), and a few attended university level education (10.6%). The majority of the participants were married (87%) and living with their families. The mean duration of hepatitis B was six years, and 72% of respondents’ family members had hepatitis B or C (Table 1).

**Table 1.** Participants’ general characteristics.

Socio-demographic variables	Number (%)	Mean (SD)
**Gender**		
Male	205 (47.5)	
Female	165 (38.2)	
Transgender Male	35 (8.1)	
Transgender Female	27 (6.25)	
**Age (years)**		48.0 (6.23)
**Education level**		
Secondary Level	228 (52.8)	
College Level	158 (36.6)	
University Level	46 (10.6)	
**Marital status**		
Married	379 (87.7)	
Unmarried	53 (12.3)	
Window	–	
**Duration of illness**		6.30 (4.71)
**Family history**		
Another member of family with hepatitis	Yes 314 (72.7) No 118 (27.3)	
**Residential area**		
Urban	198 (45.8)	
Rural	234 (54.2)	

SD = Standard deviation.

A Pearson-moment correlation was used to examine the variable relationships. The result revealed that knowledge has a highly significant positive association with self-management (*r* = 0.107*, *p* = .05), attitude has a positive association with self-management (*r* = .157), while practice has a negative association with self-management (−.04). Self-management of hepatitis has a significant positive relationship with quality of life (*r* = .217**, *p* = .01). Furthermore, there is a significant negative relationship between quality of life and self-stigma (*r* = −.145**, *p* = .01), as shown in Table 2.

**Table 2.** Pearson-moment correction among the variables.

Variables	Attitude	Practice	Self-management	Quality of life	Self-stigma	M (SD)
Knowledge	.136**	.043	.107*	.060	−.085	3.7 (2.04)
Attitude	1	.156**	.157	.005	−.118*	3.9 (2.48)
Practice		1	−.042	.063	−.016	4.16 (2.9)
Self-management			1	.217**	−.097*	1.69 (.69)
Quality of life				1	−.145**	2.94 (3.2)
Self-stigma					1	3.05 (2.1)

Note**:** Correlation is significant at **0.01 level and *0.05. M = mean and SD = standard deviation.

As shown in Table 3, gender effects were examined on the study variables. Pillai’s trace = .536, *F* (2,430) = 5.563, *p* .001, n2 = 0.66. According to the results, F values showed the differences among the variables. Males had a high level of knowledge but a low level of practice and self-management. The study also included the transgender, the most neglected population in Pakistan. In the study, transgender people showed poor knowledge, self-management, and quality of life and the highest stigmatization. Women were moderate in self-management, but they showed high stigma and low quality of life.

**Table 3.** Multivariate effect on the study variables.

Variables	Male (205)	Female (165)	Transgender (62)	f	*P*-value	Effect size
	M (SD)	M (SD)	M (SD)			
Knowledge	6.14 (2.08)	3.23 (1.61)	1.03 (0.73)	8.2**	.000	.060
Attitude	2.33 (2.3)	2.78 (2.94)	0.4 (0.02)	4.1*	.028	.011
Practice	3.47 (1.03)	5.1 (3.57)	1.43 (0.55)	2.06*	.04	.004
Self-management	2.17 (6.02)	4.21 (13.0)	2.0 (0.91)	−3.06**	.001	.068
Quality of life	3.63 (3.2)	1.34 (1.04)	0.37 (0.31)	6.21**	.000	.021
Self-stigma	2.82 (1.43)	5.13 (3.63)	3.96 (1.68)	−4.1*	.001	.081

Note**:** M = mean, *p* = significance, *p* < .001**, *p* < .05*.

The hypothesis testing result showed that knowledge has a significant positive association with hepatitis B management (B = 0.38***, *p* = .000). Attitude also has a significant positive association with hepatitis management (B = 0.15**, *p* = .001), while people have poor practices towards hepatitis management (B = −0.07, *p* = .07) (the practice has a negative association with hepatitis management). It is hypothesized that self-management leads to a quality of life. The current study found a positive relationship between self-management and quality of life (B = 0.36, *p* = .001). But the self-stigma has a negative association and decreases the quality of life of hepatitis patients (B = −0.53**, *p* = .001) (Table 4).

**Table 4.** Model assessment with interaction.

Hypothesis	Relationships	Beta	SE	t-value	*P*-value
H1	Knowledge→Self-Management	0.384	0.75	11.33	.000
H2	Attitude→Self-Management	0.153	0.54	4.32	.001
H3	Practice→Self-Management	−0.072	0.121	1.56	0.07
H4	Self-Management→Quality of life	0.36	0.44	2.03	.001
H5	SM*SS→Quality of life	−0.537	0.040	3.61	.001

SM = Self-management, SS = self-stigma.

## Discussion

**Figure 2.**
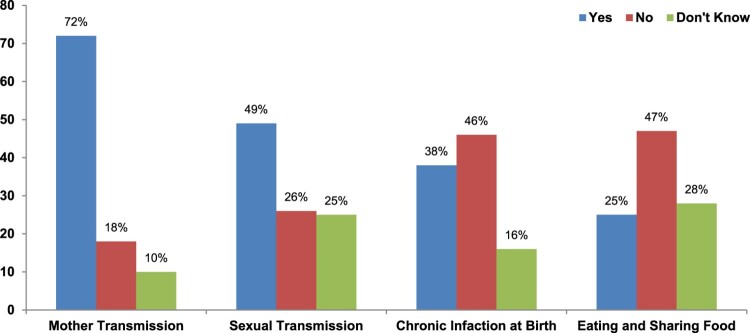
Study participants’ responses regarding transmission routes of hepatitis.

In Pakistan, hepatitis B is a major concern. Knowledge of hepatitis B facts, including transmission routes and prevention methods, is deteriorating even among hepatitis patients. The respondents moderately understood hepatitis B prevention (Figure 2). Mother-to-child transmission is the major cause of the high prevalence of chronic hepatitis in Pakistan, according to 72% of the patients. The majority of hepatitis B cases, according to 49 percent of respondents, are sexually transmitted. Only 38% of those polled were aware that the biggest risk of persistent infection arises after childbirth. Approximately 25% of respondents believed that eating with hepatitis B patients could transmit hepatitis B (Figure 2). Previous studies conducted in Vietnam at selected health clinics revealed knowledge gaps among medical students regarding hepatitis B transmission, health risks associated with hepatitis B infection, and diagnostic processes (Wang et al., 2016). The current study results revealed (Table 2) patients have a positive correlation between knowledge and hepatitis management, but they have a negative association between practice and hepatitis management. Lack of understanding and misconceptions about how hepatitis B is transmitted, as well as the higher risk of contracting hepatitis B if infected at birth, can result in missed opportunities for hepatitis B prevention and vaccine, as well as discrimination against people with hepatitis B. Similar findings have been reported in Ghana (Adam & Fusheini, 2020; Osei et al., 2019). An et al. (2021) investigation revealed limited information on hepatitis B prophylaxis. This suggests that these individuals did not receive sufficient hepatitis B prevention education. People and hepatitis patients may have been incapable of managing their own health due to inadequate hepatitis B infection prevention education. Screening healthy donors can assist in averting infection and quantifying the disease burden in Pakistan, where 1.5 million people donate blood each year (Zahoor et al., 2021). Due to poor facilities, economic status, and a lack of awareness about disease transmission (Ali et al., 2011), A lot of efforts have been made by different institutes to educate the public about hepatitis. The National Health Institute (NIH) Islamabad determined environmental factors and arranged training of lady health workers and supervisors to raise awareness of hepatitis and address preventive measures to cope with the disease (Ashraf et al., 2020).

This study found a correlation between self-management and disease awareness in chronic hepatitis B patients. The impact size score of self-management behaviors in this study (Table 4) was approximately 38% of the total score, showing that this population displayed an adequate amount of self-management behaviors (Xu et al., 2018). The relationship between proper information and better physical and mental health outcomes may increase patients’ sense of responsibility for managing their health behaviors. Consistent with previous research, we found a strong correlation between knowledge and self-management in persons with diabetes, cancer survivors, and chronic heart disease (Arda Sürücü et al., 2017). According to the practice scale (Table 4) and hepatitis management, patients in our study did not have a sufficiently positive attitude toward practices to control chronic hepatitis B, which impacted their ability to self-manage the condition. According to our data, habits are a good intervention target for patients with chronic hepatitis B. The current study found that men were knowledgeable about hepatitis B, whereas women were more concerned with protocols and management. Transgender individuals had insufficient hepatitis information, treatment, and management. This could be one of the reasons that a study found a higher prevalence of HBV and HCV among transgender individuals (Fatima et al., 2022; Moradi et al., 2022).

The current study also examines hepatitis C (C OR B) patients’ hepatitis management and quality of life. The findings demonstrated that self-management had a 36% influence on the quality of life of hepatic patients (Table 4). The current study’s findings indicated that their quality of life improves when patients can manage their disease or improve their health. Patients with Hepatitis B must finish their antiviral treatment regimen to improve their quality of life (Cortesi et al., 2020). Medication adherence improves patients’ quality of life. Patients with active hepatitis B can live healthy lives and avoid illness (Zhang et al., 2022). Antiviral therapy decreases viral load while maintaining physical health, hence boosting self-esteem and quality of life (Wang et al., 2012). Pain raises the likelihood of issues, lowering one’s quality of life. The ability to prevent sickness reduces with age, resulting in a decrease in life quality. It is easier for educated people to accept their illness. People with a higher level of education are typically more competent (Miftahussurur et al., 2020). Our study associated a three-year earlier diagnosis with a higher quality of life. People in pain may feel depression, which reduces their quality of life (Sarin et al., 2015). Inactive hepatitis surface antigen (HBsAg) carriers have the highest health-related quality of life, which may decrease as the disease progresses (Sitlinger & Yousuf Zafar, 2018). Quality of life was affected by both the duration of treatment and the routine of patient care.

**Figure 3.**
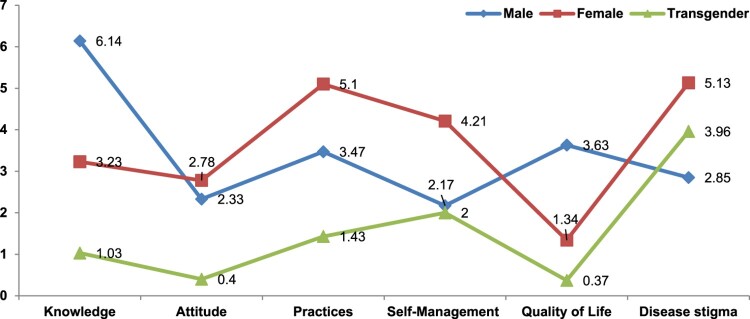
Respondents’ mean score in knowledge, attitudes, and practices towards hepatitis.

**Figure 4.**
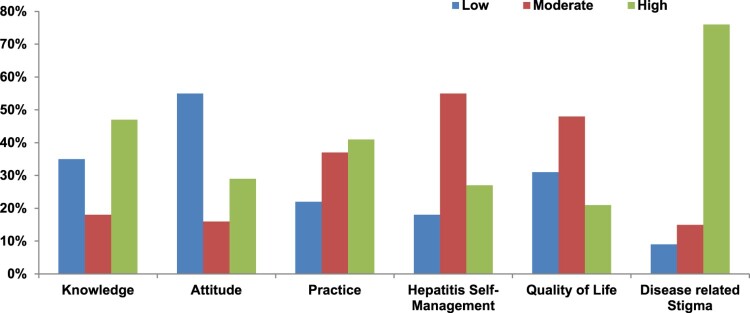
Level of knowledge, attitudes and practices, hepatitis self-management, quality of life, and stigmatization among Hepatitis B patients.

Specifically, stigmatization can negatively affect identity and mental health (Major & O’Brien, 2005). In numerous Asian nations, hepatitis stigma is poorly recognized. This study examined how stigma affects the quality of life of individuals. According to Table 4, stigma decreases life quality. In Figure 4, it is seen that respondents faced a high level of stigmatization. As a result of stigma, Hepatitis B patients have a lower quality of life because they shun individuals. According to the modified labeling theory (Link et al., 1989), discounting oneself due to discrimination based on a socially devalued attribute may have detrimental consequences (Noor et al., 2016). Self-stigma has been connected to increased symptom intensity, decreased treatment adherence, increased suicidal ideation, and a considerable decrease in quality of life (Vrbova et al., 2018). It is worth noting that quality of life indicators have been linked to people’s perceptions of their own neurocognitive impairments rather than medical professionals’ assessments of patients’ health. The widespread stigma attached to health concerns is a substantial impediment to the proper treatment and rehabilitation of sick people (Mirnezami et al., 2016). Improving the quality of life for individuals with chronic diseases necessitates determining the most efficient methods of raising awareness of the detrimental effects of stigma. Women and transgender people faced significant stigmatization (Figure 3).

## Limitations

The study has a few limitations, including the fact that data was collected from only two cities in Pakistan, which provides a valuable insight into local understanding of hepatitis, but only a small number of transgender people participated in the current study. The surveys were self-administered by hepatitis patients. The survey questions took 20–25 min to complete, decreasing answer accuracy. Because transgender people are unable to read surveys, responses were based solely on verbal responses.

## Conclusion

Most of the Pakistani hepatitis B patients have disease knowledge and its self-management, but transgender people are less informed about the disease and its management. Women patients have more practical attitudes toward self-management than men. People have a higher quality of life due to self-management, but they are concerned about being stigmatized, directly impacting their quality of life. It is critical to focus on the quality of life and stigmatization of people with chronic illnesses in terms of their human rights, dignity, and physical, mental, and social well-being. There is a need to incorporate stigma and quality of life perspectives among persons living with stigmatized conditions into national development programs (initiatives, awareness campaigns and disease-related educational activities).

## Implications of the study

Our findings have important implications for hepatitis researchers and physicians. Transgender hepatitis B positive patients should be monitored first due to poor self-management. It is essential to educate patients about complex self-management. Second, self-management activities for patients in rural areas should be regularly evaluated to identify gaps and gauge the success of patients’ education. It is also recommended to develop methods to increase patients’ social support and sense of self-efficacy. Self-efficacy education should be given to people with hepatitis to boost their confidence. In order to enhance self-management, care and interventions should include strengthening the social support systems of hepatitis patients. The clinical strategy should consider the patient’s demographics (gender and education level).

## Data Availability

The data will be provided by the corresponding author upon reasonable request.
